# Roe Deer Reproduction in Western Poland: The Late Autumn Rut Phenomenon

**DOI:** 10.3390/ani14213078

**Published:** 2024-10-25

**Authors:** Robert Kamieniarz, Michał Szymański, Magdalena Woźna-Wysocka, Bartłomiej M. Jaśkowski, Marcin K. Dyderski, Emilia Pers-Kamczyc, Maciej Skorupski

**Affiliations:** 1 Department of Game Management and Forest Protection, Poznan University of Life Sciences, Wojska Polskiego 28, 60-637 Poznan, Poland; maciej.skorupski@up.poznan.pl; 2 Forest District Łopuchówko, Łopuchowko 1, 62-095 Murowana Goslina, Poland; 1989.m.szymanski@gmail.com; 3 Department of Medical Biotechnology, Institute of Bioorganic Chemistry Polish Academy of Sciences, Noskowskiego 12/14, 61-704 Poznan, Poland; mwozna@ibch.poznan.pl; 4 Department of Reproduction and Clinic of Farm Animals, Faculty of Veterinary Medicine, Wroclaw University of Environmental and Life Sciences, Plac Grunwaldzki 49, 50-366 Wroclaw, Poland; bartlomiej.jaskowski@upwr.edu.pl; 5 Institute of Dendrology, Polish Academy of Sciences, Parkowa 5, 62-035 Kornik, Poland; mdyderski@man.poznan.pl (M.K.D.); epk@man.poznan.pl (E.P.-K.)

**Keywords:** roe deer, late rut, reproductive potential, ovary, corpus luteum, fertility decline, heat stress, global warming

## Abstract

Roe deer (*Capreolus capreolus* L.) populations in Poland are characterized by low productivity, which is why their reproductive potential was investigated. The presence of corpora lutea (CL) on the ovaries of females hunted in autumn and winter in the years 2015 and 2016 was assessed. Most animals were post-rut and most often had multiple ovulations. However, in early autumn 2015, 60% of the females had no CL. Therefore, they did not participate in mating at the turn of July/August. Those that did ovulate were found to have one CL. In late autumn, 97% of the females were post-rut, mostly with twin ovulations. This confirmed the occurrence of a late autumn rut. This phenomenon had been suggested in studies but not confirmed. In contrast, almost 100% of roe deer ovulated in summer in 2016. It was hypothesized that the reason for the low mating activity of roe deer in the summer of 2015 was heat stress and limited food resources. The summer was exceptionally hot, with many days in July and August when temperatures exceeded 30 °C. The heat combined with low rainfall led to extreme drought. Meanwhile, July and August are the months of mating activity for roe deer. The late autumn rut allowed the roe deer—a monoestrous species—to limit the consequences of a decrease in mating activity or fertility during the hot summer. Global warming may affect roe deer reproduction, so climatic conditions should be considered in population studies, not only in terms of food availability.

## Simple Summary

Roe deer (*Capreolus capreolus* L.) are a common species in Central Europe. In the 21st century, it was discovered that roe deer in Poland have fewer fawns than in the past. Therefore, their fertility was investigated. It turned out that most of the females had a chance of having twins. However, in summer 2015, 60% of females did not participate in mating, and those that did had the chance of having only one fawn. In this unfavorable year, the roe deer experienced an alternative estrus in late autumn. As a result, the decrease in the number of fawns was not very large. We have described the consequences of the late autumn estrus, which had been reported in the past but not confirmed in scientific studies. In 2016, almost 100% of the females studied mated again in the summer. We assume that the reason for the low mating activity of the roe deer in summer 2015 was heat stress, which limited the normal function of their bodies. This summer was exceptionally hot with days above 30 °C, which coincided exactly with the summer mating season of roe deer. The high temperatures were accompanied by low rainfall, leading to extreme drought and reduced food availability. Global warming may impact the reproduction of roe deer and other animals, so it is worth considering climatic conditions in population research, and not just in terms of food availability.

## 1. Introduction

The roe deer (*Capreolus capreolus* L.) is characterized by high ecological plasticity; hence it is a common species in Europe, including Poland. It inhabits mostly forest environments, but also anthropogenic environments, including open agricultural areas [1,2,3]. The occurrence of roe deer in various habitats results in differences in population density and body mass. For example, in Poland, the highest densities—10–38 ind. km^−2^—were found in small midfield forests [4,5]. In large forests, roe deer occurred less frequently—3–25 ind. km^−2^ [6]. However, the lowest density—2–14 ind. km^−2^—was characteristic of the roe deer populations inhabiting open agricultural landscapes [7]. In turn, when analyzing the body mass, it was found that roe deer from forest areas had a body mass 3–4 kg lower than roe deer inhabiting agricultural areas [6,8,9]. Smaller differences were found when analyzing the mean age of roe deer in various environments. The mean age was similar (4 years old) in both agricultural areas [7] and in large forests [6].

The population density and body mass of forest and field roe deer differed, but their reproductive potential in the 20th century was similar. At the turn of autumn and winter, the fertility of forest roe deer expressed by the number of corpora lutea (CL) on the ovaries or the number of embryos/fetuses per pregnant female was on average 1.8 [10]. The fertility in the populations of field roe deer was similar, estimated at 1.9 young per female participating in reproduction [11]. However, productivity was lower and more diversified. In the field population, an average of 0.8 young per female was recorded in the autumn population [12]. In the forest population, however, the average was 0.6 young per female [10]. In the 21st century, this level decreased and averaged 0.5 in fields and forests [7,13]. However, in some field and forest populations, there was an average of only 0.3 young per female in autumn [6,7].

Roe deer, unlike other representatives of the *Cervidae* family, are monoestrous. Females that are not fertilized will not come into heat again within the same calendar year [14,15]. Additionally, roe deer exhibit delayed embryo implantation, first described by Bischoff (1854) [16]. The consequence of this is a prolonged pregnancy. This phenomenon is common in some orders of mammals, but among wild Artiodactyla, it occurs only in roe deer [17,18]. The reproductive season in roe deer usually falls at the turn of July and August. If fertilization occurs, the embryo reaches the blastocyst development stage a few to a dozen days after ovulation. After its expansion, the blastocyst hatches from the surrounding *zona pellucida* (day 12–13 of pregnancy) and becomes bilaminar, consisting of the dorsal epiblast and ventral primitive endoderm [19]. Over the next 4–5 months, further development of the embryo is slowed down but not stopped [19,20,21]. This phenomenon, called diapause, causes the pre-implantation embryo of roe deer to reach a size of 1 mm only after about 4 months from fertilization, in contrast to, for example, the closely related ruminant farm animals, in which this period lasts a dozen or so days [22]. This is probably why, regardless of fertilization, the activity of the corpora lutea (CL) persists until the end of the diapause period [15,23,24]. In females that are not fertilized, luteolysis occurs only at the turn of December/January, which under normal conditions prevents renewed reproductive activity in one season [25]. The fact that roe deer bucks provide functional semen from June to August also confirms the absolute monestrous character of the roe deer [16]. On the other hand, the pregnancy CL, as in other ruminants, remain active until the parturition [14,17]. Maintaining the activity of the CL after the end of diapause is certainly related to embryonic signaling, which changes with the rapid acceleration of embryo growth. During this time, it undergoes fast elongation (December and January), after which the implantation and further embryonic growth occur. The young are born at the turn of May and June [19,21,23]. The female mostly gives birth to two fawns, less often one or three, exceptionally four or even five [26].

Animals are exposed to many adverse environmental factors, so they have developed various adaptive mechanisms, such as seasonal habitat changes [4,27]. Female roe deer are income breeders [28]. The rutting season and lactation, which are energy-demanding, therefore take place mainly in summer, a period of abundant food [12,29]. However, to ensure that the birth of fawns and the next lactation period coincide with another period of high food availability [28,30], roe deer use the previously described delayed embryo implantation [15].

In the 21st century, humans and animals experience high temperatures in the summer [31]. At the same time, numerous studies, both on human reproduction and farm animals (including ruminants), indicate the negative impact of temperature increase on the quality of gametes, embryo development, or embryo implantation and, consequently, the number of births [32,33,34]. For this reason, it is important to monitor the reproductive potential of animals, which depends on many factors, including the condition of the animal. In females, it is expressed, among others, by the number of ovarian follicles or CL present on the ovaries and the proportion of successful pregnancies. Roe deer mating in the summer can be a model species for such monitoring.

In the case of a hot summer, which, among other things, limits the availability of food, animals should save energy. To survive, roe deer can therefore give up expenses related to the mating season. However, the dynamics of the population are shaped by mortality, but also reproduction [35]. Mortality avoidance is beneficial. On the other hand, the lack of summer estrus in roe deer may mean a lack of offspring in a given year. Here, another adaptive mechanism probably comes to the rescue: the late autumn rut. Some researchers, starting from Bischoff (1854) [16], reported that in roe deer, ovulation may occur also in late autumn. However, this has not been confirmed in studies [19]. Meanwhile, such a situation most likely occurred during our study on roe deer fertility in the Poznań region (western Poland) in 2015.

This study aimed to assess the reproductive potential of female roe deer during a period of climatic changes over two consecutive years that differed in weather conditions.

## 2. Materials and Methods

### 2.1. Research Area and Sampling Time

**Figure 1:**
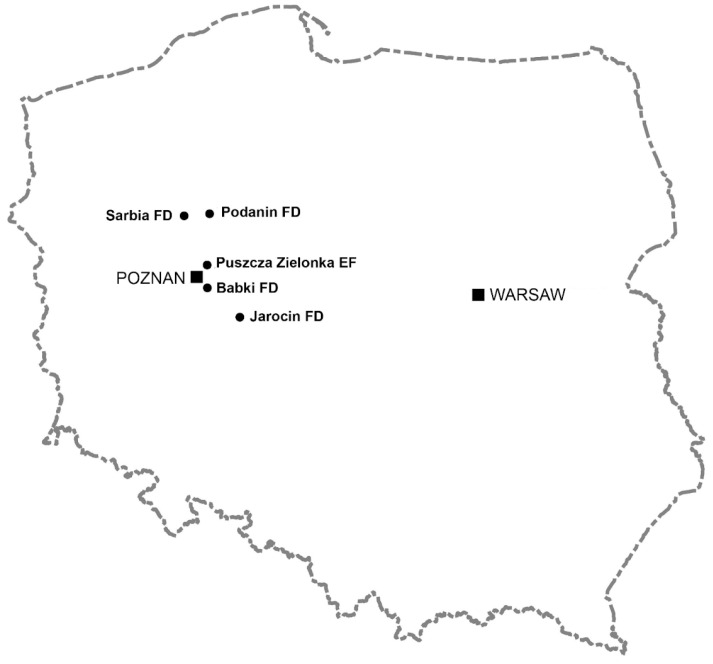
Location of areas in western Poland where the reproductive potential of roe deer was tested during the two following hunting years: 2015/2016 and 2016/2017 (Puszcza Zielonka experimental forest (EF), and Forest Districts (FDs) Babki, Jarocin, Podanin, and Sarbia).

All analyses were conducted on female roe deer harvested by hunters during two hunting years, 2015/2016 and 2016/2017. In Poland, female roe deer hunting lasts from 1st October to 15th January. We divided the hunting season into two-month periods: autumn (October–November) and winter (December–January). In Poland, these seasons usually differ in weather conditions. Animals were harvested from forests, as well as agricultural areas (Figure 1). Most of the animals (*n* = 60) were obtained from the Puszcza Zielonka—the experimental forest of the Poznan University of Life Sciences, which since 1965 has been designated for hunting research, as well as from areas with high forest cover, located to the north of Poznań (*n* = 37, Forest Districts Podanin and Sarbia). Roe deer were also harvested from agricultural areas located both far from forests and in their vicinity (*n* = 34, Forest Districts Babki and Jarocin).

### 2.2. Characterization of the Body Mass and Age of Roe Deer Female

After hunting, animals were immediately collected and analyzed. Firstly, reproductive systems were collected during the evisceration of the animals. The collected material was transported to the laboratory in stable, cold-temperature conditions for further analysis. Subsequently, body mass was weighed (with an accuracy of 0.5 kg) after the removal of internal organs from the body cavities.

Female age was assessed based on the development and wear of teeth on the prepared mandibles, according to the methodology described by Zalewski et al. [36]. Collected females of known age were grouped according to their reproductive potential into fawns (at the age of 5–8 months, usually without ovarian activity), yearlings (in the 2nd year of life, able to participate in reproduction for the first time), and older (in the 3rd year of life and above), which usually reproduce every year [37]. The older group was divided into two categories. The first group consisted of females aged 3 to 6 years, usually reproducing every year, and the second consisted of individuals older than 6 years—not always able to reproduce every year [11].

### 2.3. Characterization of Female Reproductive Potential

The reproductive potential of the females was evaluated based on the reproductive system and ovarian activity, on which the presence and numbers of corpora lutea (CL) and/or follicles were assessed. The analysis of the CL presence as proof of successful ovulation and its persistence regardless of fertilization in a particular hunting year [14,15] was of the highest importance for us. We treated females as reproductively active individuals when CL were present on the ovaries. The presence of dominant or growing ovarian follicles suggested reproductive activity at a later period.

### 2.4. Uterus Analysis

**Figure 2:**
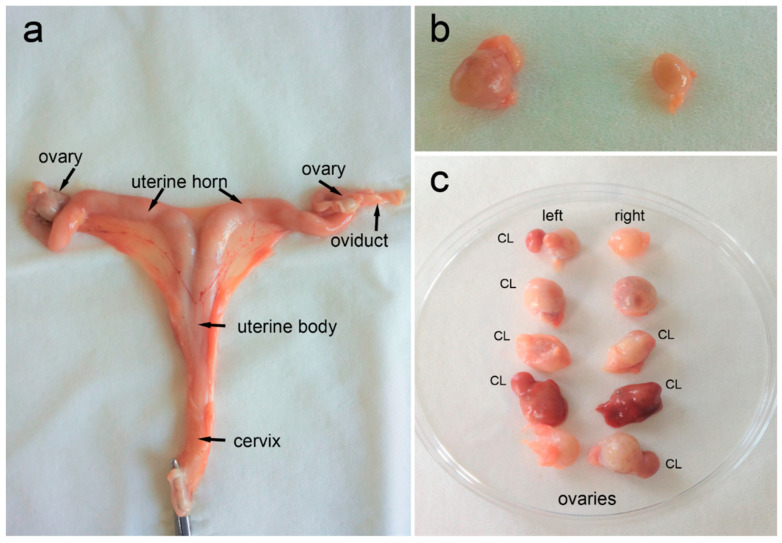
Female roe deer reproductive system. (**a**) Overview of a reproductive tract after partial separation from the *ligamentum latum uteri* and all the other surrounding connective tissues, (**b**) ovaries with numerous small or growing ovarian follicles but without corpora lutea (CL), and (**c**) ovaries with the presence of CL.

Each reproductive system was analyzed individually. Laboratory tests included macroscopic assessment and morphometric measurements of the reproductive system of females. Before measurements were taken, each uterus was thoroughly prepared by precise separation from the *ligamentum latum uteri* and all the other surrounding connective tissues. The uterine measurements taken included the mass, length of the cervix (distance between the external and internal orifice of the uterus) and uterine body (distance between the internal orifice of the uterus to bifurcation), length (distance between bifurcation and the oviduct), width (measured directly after separation of both horns), and thickness of the uterine horn wall (measured in cross-section), and length of the oviducts (from the end of the horn to the ovary). The measurements were performed using a laboratory caliper, always in the same and precisely defined places (see Figure 2). The length of the oviducts was measured after separation from the surrounding connective tissue and straightening. The final uterine mass with oviducts was determined using an analytical balance after the ovaries were cut off and the lumen of the uterus was emptied (in the case of pregnancy presence). The number and size of embryos/fetuses were assessed in the pregnant females after the end of diapause. However, the reproductive organs of females with visible pregnancy were excluded from the final morphometric analysis.

This study included only physiological uteri, which did not present pathological/degenerative changes in the macroscopic assessment after longitudinal incision of the uterine horns. This evaluation involved a detailed visual inspection of the uterine lumen for any abnormal thickening of the endometrium, cysts on its surface, or accumulation of purulent secretions.

### 2.5. Ovarian Activity

Each ovary was inspected individually and qualified into the following categories: 1–3 = no CL and (1) ovary without activity (lack of ovarian follicles), (2) ovary with the presence of numerous small ovarian follicles, (3) ovary with the presence of growing or dominant follicles (with diameter > 1 mm); (4) ovary with the presence of CL. Moreover, the diameter of each growing or dominant follicle, as well as CL present on the ovary (both left and right sides), was measured.

Each time an ovary was assessed, its length (distance between *extremitas tubaria* and *extremitas uterina*), width (distance between *margo mesovaricus* and *margo liber*), and thickness (distance between *facies medialis* and *facies lateralis*) were measured using a laboratory caliper.

### 2.6. Climate Conditions

Meteorological data were obtained from the Institute of Meteorology and Water Management—National Research Institute (http://danepubliczne.imgw.pl/data/dane_pomiarowo_obserwacyjne/ accessed on 26 August 2024) and then processed by the authors. The data for the Poznań station were downloaded using the climate package [38]. Standardized Precipitation Evaporation Index (SPEI) data were obtained from the Global SPEI database [39,40]. Data describing climate conditions from the nearest weather station to the hunting location were downloaded. Therefore, data characterizing Puszcza Zielonka experimental forests were collected from IMG station Poznan-Ławica, for Podanin and Sarbia Forest Districts from IMG Chrząstkowo, for Jarocin Forest District from IMG Jarocin, and for Babki Forest District from IMG Kórnik station. The following information was collected from the database: average daily air temperature (°C), maximum air temperature (°C), and monthly precipitation (mm). According to the obtained meteorological data, we also described the number of days with an air temperature higher than 30 °C for the month preceding the summer roe deer estrus period (June) and for the following months until the end of the year.

**Figure 3:**
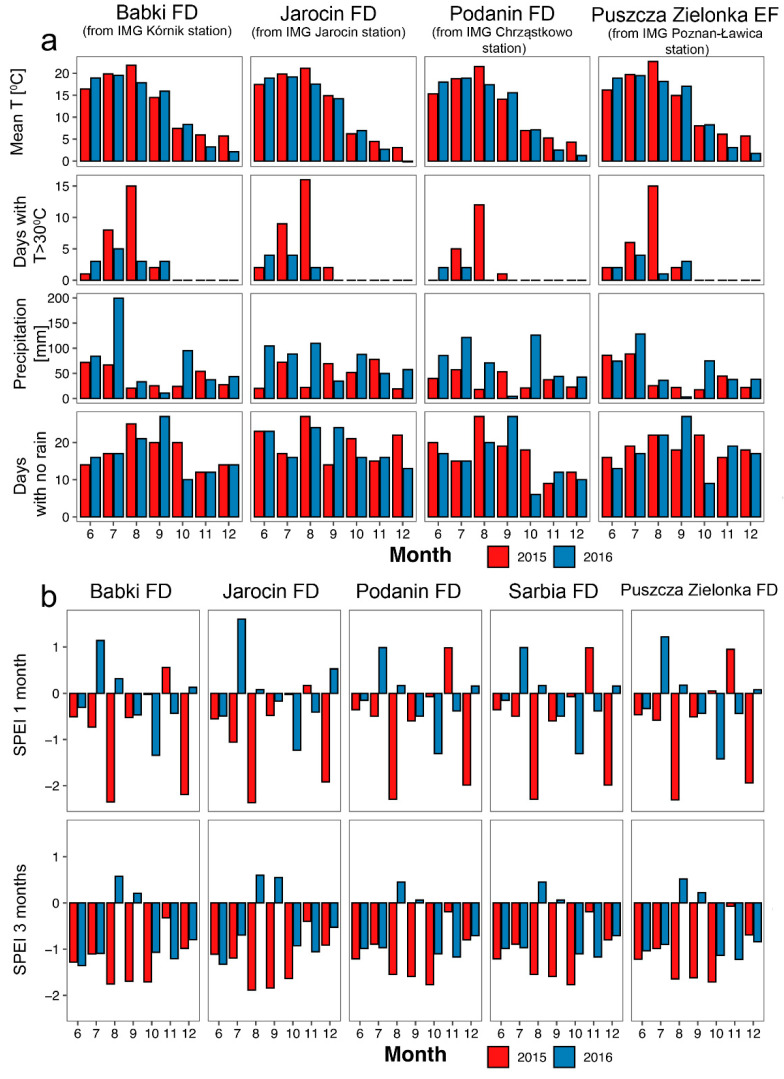
Comparison of climate data from four meteorological stations closest to studied areas (**a**) and Standardized Precipitation Evapotranspiration Index, acquired from the gridded dataset for studied areas (**b**). For (**a**), we obtained data from the nearest available meteorological station, i.e., IMG Kórnik station for Babki Forest District (FD), IMG Jarocin station for Jarocin FD (both within the forest inspectorates), IMG Chrząstowo station for Podanin (~40 km) and Sarbia (~60 km) FDs, and IMG Poznań-Ławica station for Puszcza Zielonka Experimental Forest (EF) (~20 km). Months—from June (6) to December (12). Sources: (**a**)—Data from the Institute of Meteorology and Water Management—National Research Institute (http://danepubliczne.imgw.pl/data/dane_pomiarowo_obserwacyjne/ accessed on 26 August 2024), processed by the authors, with data for the Poznań station downloaded using the climate package [38]; (**b**)—the Global SPEI database [39,40].

The studied years differed in climate characteristics, but the trend was consistent among studied areas (Figure 3a). Although mean monthly temperatures were similar in summer 2015, July and August 2015 had more days with maximum temperatures exceeding 30 °C, lower precipitation (especially in August), and a slightly higher number of days with no rain, compared to 2016. These conditions resulted in severe drought, expressed by negative values of the Standardized Precipitation Evapotranspiration Index (SPEI; Figure 3b). In August 2015, values of 1-month SPEI were below −2, indicating an extremely dry period, while in August 2016, they exceeded 1, indicating moderate wet conditions. Values of 3 month SPEI were less extreme but also showed a similar trend.

### 2.7. Data Analysis

We used R software [41] version 4.3.2. for data analysis. We assessed the differences in morphometric characteristics of roe deer reproductive organs using redundancy analysis (RDA), a constrained version of principal components analysis. This means that RDA shows reduced dimensions of the multivariate dataset and tests the fitness of independent constraint variables (body mass, age, season, year, and season × year interaction). We used the vegan package [42] for RDA. Prior to RDA, we centered and scaled all variables (subtracting the mean and dividing by standard deviation). We ensured a lack of artifacts in RDA by visually inspecting the dispersion of points within the ordination space and using diagnostic screeplots. We tested the significance of the constraints by reducing the model based on AIC, as well as using the PERMANOVA test, i.e., permutation-based ANOVA-like test of significance.

We used Generalized Linear Mixed-Effects Models (GLMMs) to assess age, body mass, season, year, and season × year interaction effects on roe deer CL and follicles. We developed GLMMs using the glmmTMB package [43]. We assumed binomial distributions of the presence/absence of CL and follicles. We initially tested GLMMs with Poisson distributions for the number of CL and follicles and checked zero-inflation and dispersion using the formal tests implemented in the DHARMa package [44]. For follicles, we did not detect any problems with dispersion, and thus, we used the Poisson distribution; however, for CL, we found statistically significant underdispersion, and therefore, we used the COM-Poisson distribution [45], designed to deal with this kind of data. Then, for each model, we selected predictors based on Akaike’s Information Criterion, corrected for small sample size (AICc), using a model with the lowest AICc as the final model. We also reported the AICc of the null (intercept-only) model to show the decrease in AICc compared to random expectation. In all GLMMs, we included an area as a random intercept to cover spatial dependency among studied areas. For models, we provided values of marginal (R^2^_m_) and conditional (R^2^_c_) coefficients of determination. They provide information about the amount of variance explained by fixed effects only and both fixed and random effects, respectively [46]. We selected models using AICc and calculated R^2^_m_ and R^2^_c_ using the MuMIn package [47]. We presented the results of the models using marginal means (for categorical predictors) and marginal responses (for continuous predictors), i.e., mean predicted value for a particular predictor assuming all other predictors at a constant level, and excluding random effects (response for a global population). For marginal means, we used the emmeans package [48], and for marginal responses, the ggeffects package [49].

## 3. Results

### 3.1. Number and Age of Collected Animals

In total, 131 individuals were collected over two consecutive years. In the 2015/16 hunting year, reproductive organs were collected from all age-class females, including fawns (eight individuals). However, since the reproductive organs of the fawns were very small (e.g., the uterus weighed an average of 4.12 g (SD 3.04)), and no visible ovarian follicles were noted on collected ovaries, these individuals were excluded from further analysis and were not collected in the following hunting year. The lack of visible ovarian follicles indicated that the fawns did not participate in reproduction. Consequently, all subsequent analyses were conducted on 123 individuals, of which 58 were obtained in 2015 and 65 in 2016. We analyzed 29 yearlings, with 15 collected in 2015 and 14 in 2016, and 94 females older than one year, with 43 collected in 2015 and 51 in 2016.

### 3.2. Morphometric Analysis of the Reproductive System

The morphometric analysis included all non-pregnant yearlings or older females (*n* = 107). The results were presented for the entire population, taking into account the division into age groups corresponding to different periods of reproductive activity (Table S1). The average uterus was characterized by a cervical length of 42.1 mm, similar for all age groups (*p* > 0.05). The average length of the uterine body was 51.05 mm, and these values differed between groups. Yearlings (who had no offspring yet) had shorter uterine bodies (44.46 mm) compared to 3- to 6-year-old females (53.4 mm; *p* < 0.05). Although the small sample size of the oldest females (>6 years) did not allow them to be distinguished from the other groups, the length of the uterine body in numerical values for the oldest females was the highest (54.31 mm; *p* > 0.05). The mean parameters of the length, width, and thickness of the uterine horns were 106.34 mm, 9.11 mm, and 1.74 mm for the right horn and 107.64 mm, 9.12 mm, and 1.72 mm for the left horn, respectively. These values for both horns were very similar. The length of the right horn changed with age; in the youngest females, its length (94.16 mm) was smaller, but only in comparison with the oldest females (107.86 mm; *p* < 0.05). The length of the left horn did not differ significantly between groups. Similarly, no differences were found between groups in the width of the right horn and the thickness of the left horn wall (*p* > 0.05). The width of the left horn depended on the age of the females; the youngest animals showed lower values (7.7 mm) than the older animals (9.55 mm and 10.57 mm for females aged 3–6 and >6 years, respectively). The thickness of the right horn wall differed between the groups of the youngest females (1.51 mm) and those aged 3–6 years (1.81 mm; *p* < 0.05), while the values for both groups did not differ from the values in the oldest group (2.06 mm; *p* > 0.05). The uterine weight for the entire population was 25.1 g, and these values for the youngest females (20.73 g) differed from the other groups (26.07 g and 32.43 g for females aged 3–6 and >6 years, respectively; *p* < 0.05). The mean length of the oviducts for the entire population was 89.07 mm and 89.2 mm for the right and left oviducts, respectively. There were no differences in oviduct length between groups.

The average size of the right ovary for the entire population was characterized by the following parameters: 10.27 mm, 7.77 mm, and 5.66 mm in length, width, and thickness, respectively. While the length and thickness of the right ovary did not differ between groups, the width was different in all groups studied; the lowest values were described for young animals (7.04 mm), intermediate in the age group 3–6 years (7.9 mm), and the highest in older females (9.15 mm; *p* < 0.05). The average weight of the right ovary was 0.53 g and did not differ between groups.

The mean size of the left ovary for the entire population was characterized by the following parameters: 10.07 mm, 7.83 mm, and 5.72 mm in length, width, and thickness, respectively. None of the described size parameters of the left ovary differed between the groups (*p* > 0.05). The average weight of the left ovary was 0.48 g. These values differed between the youngest females and those aged 3–6 years (0.39 g and 0.5 g, respectively; *p* < 0.05), while they did not differ between these two groups and the group of the oldest females (0.58 g; *p* > 0.05).

**Figure 4:**
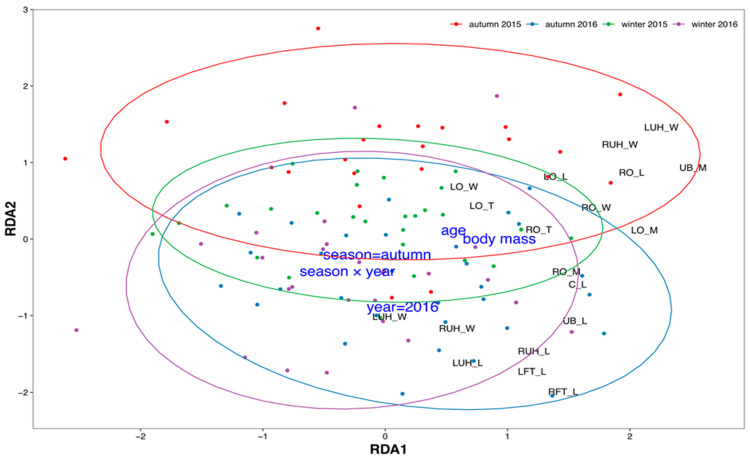
Results of redundancy analysis of roe deer reproductive system morphometric characteristics: points represent individuals, colored according to season and year; black labels indicate scores of particular morphometric characteristics; and blue labels represent environmental constraints (season, year, body mass, age, and their interaction, see Table 1). Ellipses represent 95% confidence areas for each group of year and season. Abbreviations: C_L—cervical length; LFT_L—left fallopian tube length; LO_L—left ovary length; LO_M—left ovary mass; LO_W—left ovary width; LUH_L—left uterine horn length; LUH_T—left uterine horn thickness; LUH_W—left uterine horn width; LOCL_N—number of corpora lutea on left ovary; ROCL_N—number of corpora lutea on right ovary; LOF_N—number of ovarian follicles on left ovary; ROF_N—number of ovarian follicles on right ovary; RFT_L—right fallopian tube length; RO_L—right ovary length; RO_M—right ovary mass; RO_T—right ovary thickness; RO_W—right ovary width; RUH_L—right uterine horn length; RUH_T—right uterine horn thickness; RUH_W—right uterine horn width; UB_L—uterine body length; UB_M—uterine body mass.

Multidimensional variability of morphometric characteristics depended on all hypothesized factors: body mass, age, season, year, and season × year interaction (Figure 4).

Constraints (body mass, age, year, season, and their interaction) explained 20.0% of total variability, and the first two unconstrained axes explained 16.9% and 12.6%, respectively. All constraints were statistically significant (Table 1). RDA revealed two main gradients of variability. The first axis (RDA1) was related to body mass and age and showed morphometric characteristics that exhibited the strongest correlation with these two variables. The second axis (RDA2) differentiated roe deer from winter and autumn, as well as between the two studied years. The most distinct were roe deer from autumn 2015, differentiated in the upper part of the ordination space. No morphometric characteristics were found to differentiate solely along the RDA2 axis. However, some increased along both axes. For example, the length of fallopian tubes and the width and length of both uterine horns increased with age and body mass but were also higher in autumn and 2016.

### 3.3. Presence of Corpora Lutea

In total, 109/123 (88.6%) roe deer had CL when harvested by hunters, with 45/58 (77.6%) in 2015 and 64/65 (98.5%) in 2016 (Table 2). The absence of CL, meaning the absence of ovulation, was observed almost exclusively in females hunted in the autumn of 2015 (12/20, 60%). In the remaining seasons, the absence of corpora lutea occurred only twice, exclusively in yearlings.

The absence of CL in autumn 2015, meaning the absence of ovulation in summer, was found in both yearlings (4/7, 57%) and older individuals (8/13, 62%). The average body mass of females participating in reproduction in the summer of 2015 was similar to those that did not show heat at that time (mean difference = 0.42 ± 0.95 kg, t = −0.44, *p* = 0.66). This was the case both among yearlings (15.3 vs. 15.1 kg) and older females (18.2 vs. 18.6 kg).

In autumn 2015, almost all roe deer were characterized by the presence of only one CL per female (seven individuals (ind.); three 2-year-olds, one 3-year-old, one 4-year-old, and two 8-year-olds) while only one individual, a 6-year-old female, had two CL. In winter 2015, one CL was observed in 7 ind., two CL in 26 ind., and three CL in 4 ind. However, from one to three CL were present on ovaries in both periods of the 2016 hunting year, and the frequency was similar between them (in autumn 2016: 6 ind. with one CL, 21 ind. with two CL, and 4 ind. with three CL; in winter 2016: 6 ind., 25 ind., and 2 ind., respectively). Overall, low reproductive activity and fertility were observed in females in the summer of 2015 (Table 2).

**Figure 5:**
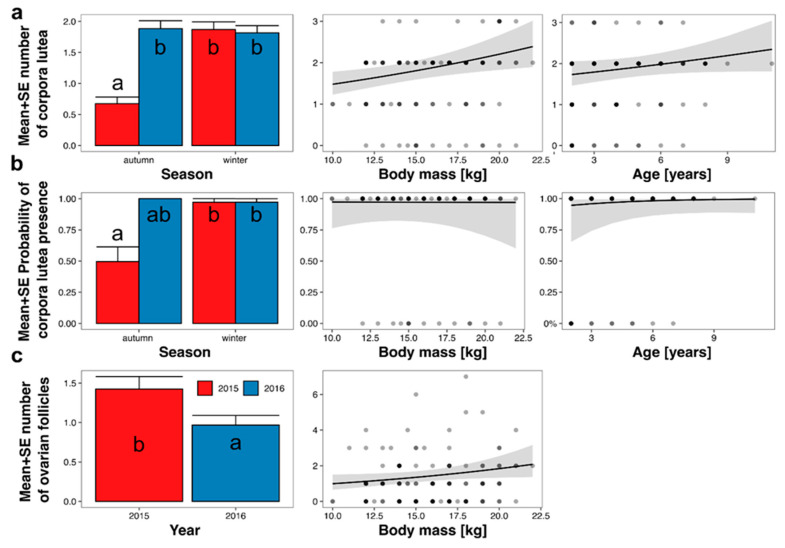
Marginal responses of the number of corpora lutea CL (**a**), probability of CL presence (**b**), and number of follicles (**c**) to predictors from final models (Table 3), calculated from GLMMs. Lines indicate marginal response (i.e., prediction without random effects and assuming all other predictors at a constant level); grey area—range of standard error; and points—observed values. Letters on bars indicate the results of Tukey’s posteriori test: groups denoted by the same letter did not differ at *p* = 0.05.

The mean number of CL on ovaries depended on age, body mass, season, and year (Table 3; Figure 5a). The effect of age was statistically insignificant (*p* = 0.08), and the effect size was low. The effect of body mass was statistically significant, and an increase in body mass from 10 to 22 kg increased the predicted number of CL from 1.5 to 2.4 (Figure 5a). The mean predicted number of CL in autumn 2015 was the lowest (0.7 ± 0.1) and differed from all other periods (1.8 ± 0.1 to 1.9 ± 0.1). The probability of CL presence depended on age, body mass, season, and year (Table 3; Figure 5b); however, only the effect of the season was statistically significant. The effect of year was statistically insignificant as all roe deer in autumn 2016 had CL on ovaries. The mean predicted probability of CL presence was 0.50 ± 0.12 in autumn 2015, 1.00 ± 00 in autumn 2016, and 0.97 ± 0.03 in winter of both 2015 and 2016.

### 3.4. Presence of Ovarian Follicles

The number of growing or dominant follicles (with a diameter > 1 mm) depended on body mass and year (Table 3; Figure 5c). The number of follicles increased with increasing body mass, from 1.0 in 10 kg roe deer to 2.1 in 22 kg roe deer. The mean predicted number of follicles in 2015 was 1.4 ± 0.2, while in 2016, it was 1.0 ± 0.1. The probability of follicle presence was not explained by any of the hypothesized predictors (the null model had the lowest AICc; Table 3).

In 2015, the absence of corpora lutea on the ovaries was noted in 13 out of 58 females, and in 2016, only in 1 out of 65 examined females. Among them were 12 individuals (yearlings and older) hunted in early autumn of 2015. These females could hypothetically reproduce in late autumn that year. The characteristics of their ovaries are included in Table S2, and the occurrence of different categories of ovarian follicles with regard to the age of the females is presented below. Of these, one yearling had no visible ovarian follicles, three individuals (3, 5, and 6 years old) were characterized by the presence of numerous small ovarian follicles, and eight individuals (three 2-year-olds, one 3-year-old, two 4-year-olds, one 5-year-old, and one 7-year-old) were characterized by the presence of more than one ovarian follicle with a diameter > 1 mm. Of these, five females had ovarian follicles > 3 mm. In addition, in winter 2015, one yearling without CL was characterized by the presence of ovarian follicles with a diameter > 1 mm on the ovaries. In 2016, the absence of CL was observed in only one yearling, in which no visible ovarian follicles were present.

### 3.5. The Occurrence of Pregnancy

**Figure 6:**
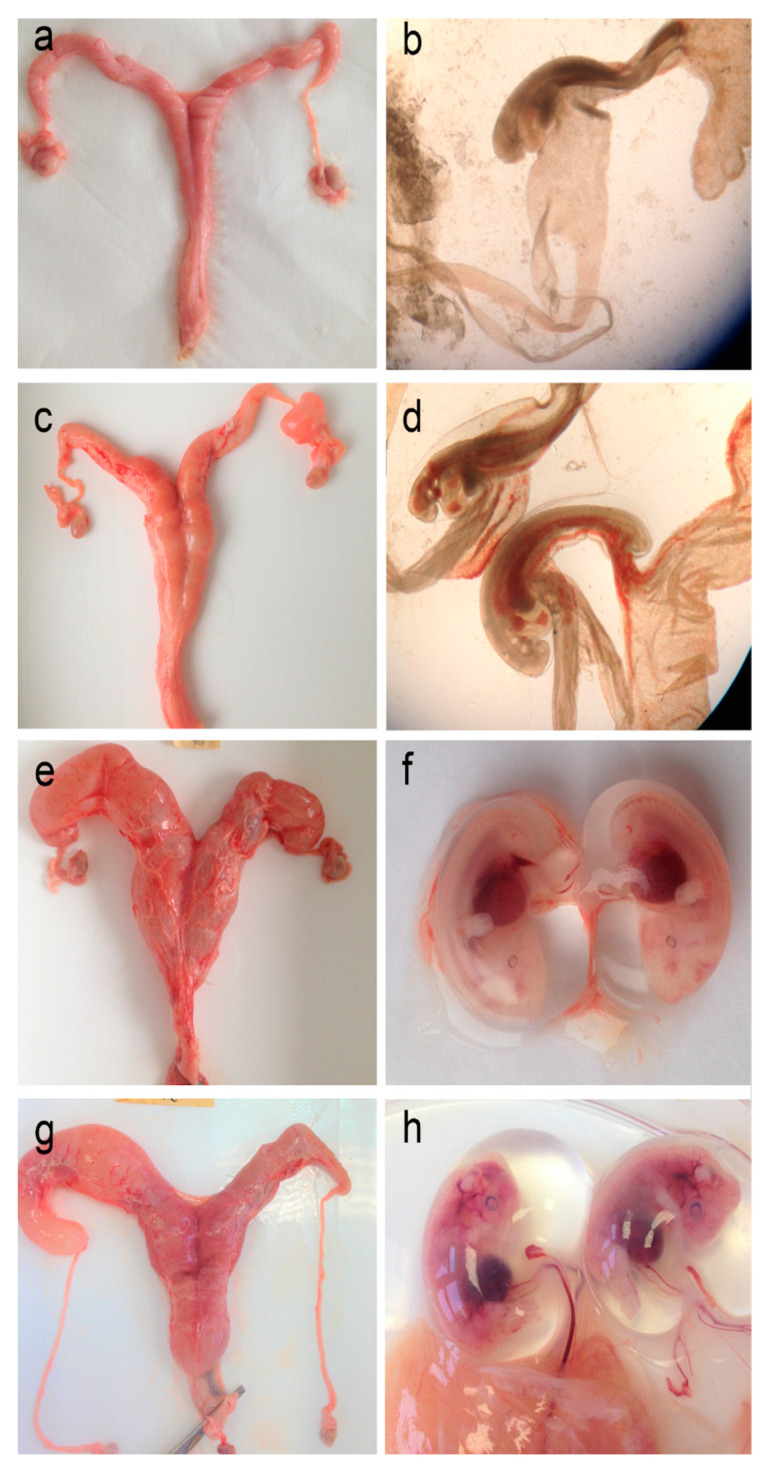
Roe deer female reproductive system and embryo overview. Representative macroscopic images of the uterus (**a**,**c**,**e**,**g**) and embryos (**b**,**d**,**f**,**h**) at various stages of development were collected from pregnant females in January 2016. The uterus of (**a**,**b**) a 4-year-old female, with two small embryos, both ~3.5 mm in length; (**c**,**d**) a 7-year-old female, with two embryos, both ~6.7 mm in length; (**e**,**f**) an 8-year-old female, with two embryos, ~18.9 and 17.2 mm in length, and (**g**,**h**) an 8-year-old female, with two embryos, both ~31 mm in length.

Among all the examined females, fetuses or embryos were found in the uterine lumen in 16 cases. The earliest observed pregnancy was found in a female shot on 21 December 2015. The largest of all the examined pregnancies was observed on 27 December 2016—the single fetus found was 66.02 mm in crown–caudal length. The smallest of the embryos was 2 mm in length (4 January 2017). In many females with early pregnancies, embryo size disparities were found, indicating that the termination of diapause may occur independently in each embryo. No such differences were found in the case of fetuses. All pregnancies were detected in individuals with two CL, and most (13 of 16) were twins (Figure 6). Of the multiple pregnancies, only one was monozygotic; the rest were heterozygotic, with embryos/fetuses located one in each uterine horn, even if multiple ovulations took place on one ovary. In two cases, the pregnancy was a singleton, and in another, the absence of the second embryo could have resulted from damage to one of the horns during evisceration.

## 4. Discussion

Reproduction is an important characteristic of a population, as it determines its dynamics and, consequently, its persistence in an area [35]. Roe deer populations are characterized by high productivity due to high fertility; thus, they usually give birth to twins [50].

The roe deer is a species that has been present in Europe for at least 600,000 years and was one of the most common mammals in the Late Quaternary period [51]. In the early 2000s, it was the most numerous deer species in many regions of Europe; hence, its hunting bag amounted to 2.7 million individuals. Consequently, it is of great economic, cultural, and ecological importance. Therefore, roe deer research programs are essential for the sustainable use of this species [52]. Studies of the reproductive systems (especially ovaries) of females shot during hunts allow for the systematic monitoring of reproductive potential [53]. Meanwhile, this trait—along with the survival of the young—determines the productivity of the population [35,54].

The litter size of roe deer depends on latitude, body mass, and density [50]. Therefore, the fertility in Norway—at high latitude and body mass (Bergmann rule), was as high as 2.0–2.4 young per female [55]. As it was a predator-free area, productivity in the autumn averaged 1.75 young per female [56]. At the same time, population productivity in mid-latitude France ranged between 0.9 and 1.3 young per female. It was also inversely proportional to the density, which was between 5 and 25 roe deer/km^2^ [57]. For this reason, productivity in Italy—low latitude and body mass—remained at the level of 0.75 (±0.4) young per female at a very high density (53.8 ± 4.8 roe/km^2^) [58]. However, during our research period in western Poland—in the mid latitudes—the fertility of roe deer populations (1.8–1.9 CL per female) corresponded to the average of European conditions [46]. On the other hand, productivity was low and probably related to female body mass. In a field roe deer population (5.3–7.2 individuals/km^2^, the eviscerated body mass of female averages 18.6 kg), productivity was 0.8–1.2 fawns per female in autumn [59]. In contrast, productivity in the Puszcza Zielonka Experimental Forest (2.2–4.8 individuals/km^2^, 14.5 kg body mass of females on average) was only 0.2–0.3 fawns per female [6]. The number of born fawns decreases with the deterioration of female condition as well as senescence [60]. However, the productivity of the population also depends on the survival of fawns. This is influenced by the quality of the environment, including food availability and weather conditions [61,62,63], as well as by predation, mainly by red foxes [56,59,64,65,66,67].

The main rut period for roe deer takes place at the turn of July and August. In females participating in reproduction, ovulation occurs from one or both ovaries [3,19]. Our studies conducted in Greater Poland (western Poland) during the hunting seasons of 2015/2016 and 2016/2017 showed that even if multiple ovulations occurred in one ovary, twin fetuses/embryos were placed one in each uterine horn.

The wall of a rupturing follicle transforms into a corpus luteum under the influence of the luteotropic mechanism. In roe deer, embryonic diapause occurs, and the corpus luteum is preserved until the implantation of the embryo [23,24]. We found differences in many females with early twin embryos, indicating that the end of diapause may have occurred independently. In the fetuses, these differences diminished. We confirmed pregnancy as early as December 21.

Corpora lutea persist until the end of diapause in both pregnant and non-fertilized individuals in a given year [14,15]. However, it should be noted that the number of corpora lutea does not necessarily correspond to the number of embryos [15,53]. In our studies, only single embryos/fetuses were found in the uterus of 2 of the 16 females that were pregnant after double ovulation. At the same time, there was probably only one monozygotic pregnancy, but with an ovulation of two CL.

The absence of estrus in roe deer usually occurs in young individuals and/or those in poor condition [37]. However, in the autumn of 2015 (October and November), it was found that 12 out of 20 examined females did not have corpora lutea on their ovaries. In our study, 4 out of 6 yearlings and as many as 8 out of 14 individuals aged two years and older did not ovulate. At the same time, the average body mass of females after ovulation did not differ from the average of individuals that did not ovulate in early autumn. In the following months, the picture of reproductive activity and fertility changed drastically. In the same research areas, corpora lutea were found on ovaries in almost all females (37/38, missing in 1 yearling). This indicates that estrus in roe deer in western Poland in 2015 also took place in the autumn.

The fact that roe deer—a monoestrous species—have an alternative mating season at the turn of November/December was first mentioned by Bischoff [16]. However, Rüegg and Ulbrich [19] stated in their review on diapause in roe deer that this was not confirmed in studies. Based on our observations, we can conclude that an alternative estrus took place in Greater Poland in 2015—most likely in November and early December. In this context, designating the alternative mating season in roe deer as late autumn estrus is justified.

Thanks to the alternative rut in the autumn of 2015, the roe deer population maintained a relatively high reproductive potential. Females that were hunted in early autumn, and thus ovulated in summer, generally had one corpus luteum. However, in December and January, the proportion of individuals with multiple ovulations, mainly twins, increased significantly. The autumn rut also allowed those females that did not ovulate in summer to participate in reproduction. Many females hunted in early autumn had no CL, but growing or even dominant ovarian follicles. Both yearling and older females had growing or dominant ovarian follicles in the autumn. Only three roe deer shot in the autumn had numerous small ovarian follicles, and only one yearling showed no ovarian activity.

The absence of the summer rut in female roe deer was not repeated in the following year. In the same control areas, all 31 deer examined had CL in October and November 2016. In December and January, corpora lutea were found in 33 of 34 roe deer. Only one yearling did not ovulate, in which no ovarian follicles were observed in January. This yearling probably did not participate in reproduction due to its very poor condition (13 kg). At the same time, the proportion of multiple pregnancies was high in both periods of 2016, which is typical for this species [26].

The absence of ovulation in many roe deer in the summer of 2015 was observed in the experimental area in the Puszcza Zielonka forest, among others. The condition of the roe deer there decreased in the 21st century but did not differ significantly between 2015 and 2016 [6]. In 2016, only the previously described yearling in very poor condition did not participate in reproduction there. The presence of summer and autumn estrus—exclusively in 2015—was observed not only in roe deer in large forests but also in fields (areas south of Poznań, Figure 1). The average body mass of females there was 18.7 kg and was higher than that in areas with a high proportion of forest (Puszcza Zielonka EF and research areas north of Poznań), where females weighed 14.8 kg on average. It should be noted that our analyses also showed no differences in the body mass of roe deer after ovulation and those that did not ovulate in the summer of 2015—both in the group of yearlings and in older females. In such a situation, it is reasonable to conclude that the direct cause of the lack of summer mating activity of many females in 2015, which was not repeated in 2016, was not body mass but the body condition of the animals during the mating season. Forest cover, an important element of roe deer habitat structure, is also unlikely to have played a decisive role. The observed delay in reproductive activity was most likely caused by an environmental factor present throughout the region that leads to periodic changes in the body function of females. The results of the morphometric measurements of the reproductive system in the autumn of 2015 differed from those obtained in the winter of the same year and the two periods studied in the following year. This result takes into account the main gradient of variability, related to age and body mass.

One of the reasons for the lack of reproductive activity of many roe deer in 2015 was probably particularly unfavorable weather conditions. Heat waves in July, especially in August, and a lack of rainfall (Figure 3a) led to a low Standardized Precipitation Evapotranspiration Index (SPEI) (Figure 3b). This leads to extreme drought [7]. Roe deer are ruminants but have exceptionally small stomachs. Therefore, they select their food [68] and have 8 to 12 feeding cycles per day [54].

The availability of optimal food resources is reflected in the condition of an individual at a given time. The condition of animals influences both survival and reproduction [69]. It should be remembered that according to the resource allocation theory, energy is expended on the growth, protection, and reproduction of individuals. In addition, the energy requirements of female mammals increase in the first month after birth. In female ungulates, this increase is between 65 and 215% (after [69]). This can lead to a negative energy balance, which contributes to a reduced activity of gonadotropin release [70]. Among other things, the literature points to a decline in reproduction in female red deer with poor body condition [71].

Numerous reports indicate that not only poor body condition but also high temperatures significantly and negatively affect the reproductive potential of animals (see rev. [33,34,72]). In our studies, we have demonstrated the occurrence of delayed estrus in female roe deer in 2015 based on seasonal observations. This could be the result of prolonged exposure to high temperatures during the summer, especially on many hot days with temperatures above 30 °C. This is supported by the absence of estrus in some females during the hot summer, while it was present in all individuals the following year when the heat occurred outside the summer mating season of the roe deer [73].

Global warming is indicated as a factor limiting fertility by raising body temperature above the physiological homeothermic point (heat stress). Animals can adapt more or less to high temperatures. To survive under prolonged heat stress, they curtail other activities, including reproduction (rev. [34]). Based on our studies, it can be assumed that female roe deer exposed to heat stress during the summer mating season experienced an alternative estrus in late autumn. In cattle, prolonged high summer temperatures significantly disrupt the physiology of the sexual cycle [74,75]. In some animals, it only normalizes 40–60 days after the end of the high-temperature period [76]. The effects of heat stress depend on the developmental stage of ovarian follicles, which grow in waves in ruminants, including roe deer. For example, heat stress in cattle can affect the early growth phase of the follicular wave when acting on early antral follicles (0.5–1 mm diameter), or it can reduce follicle size and estradiol production and cause attenuation of dominance in large non-dominant follicles if it acts on the dominant follicle phase (10–15 mm diameter), or it may impair follicular cooling and increase ovulation failure if it acts on pre-ovulatory follicles (>15 mm) (rev. [32]).

We observed a high proportion of female roe deer with delayed estrus after summer 2015 with a large number of hot days (air temperature > 30 °C). At the same time, we found that the morphometric parameters of the reproductive system of female roe deer in autumn 2015 differed from those observed during other study periods. In 2015, the number of ovarian follicles in the studied females was also higher than a year later. At the same time, roe deer that ovulated in the summer of 2015 had reduced fertility (1.1 CL per female participating in reproduction). All of this indicates the impact of persistently high temperatures on the sexual cycle, including the maturation of oocytes in roe deer.

The roe deer is a species classified as an income breeder. It has a rut in the summer, which is a period of rich food abundance. To ensure that the young are born at the beginning of the next summer, the animals slow down the embryonic development of the embryo. The extension of the active pregnancy period through embryonic diapause allows for mating and the birth of young at optimal times for this species [18]. Embryonic diapause has been described more extensively since the second half of the 20th century [14,17,23] and is also being studied contemporarily thanks to new techniques [15,19,20,21]. In addition, the roe deer, a monoestrous species, may not ovulate during the summer heat and/or drought, instead having an alternative mating season in the autumn. In this way, it avoids a pause in reproduction. Additionally, females that ovulated in the autumn of 2015 were more likely to have twins, which is typical for this species. On the other hand, individuals that participated in the rut during the hot summer could only expect a single offspring in the following year. This is probably the reason why the productivity in the Puszcza Zielonka Experimental Forest (monitored for a long time) was particularly low after the unusual mating season in 2015 [77].

This work has shed new light on a mechanism that has, so far, only been hinted at in the scientific literature. Climate change, resulting in hot and dry summer periods, may cause delayed rutting in roe deer to occur more frequently. This will allow for a deeper understanding of the phenomenon. Therefore, as a supplement, we have included morphometric data collected during our research, which illustrate the unusual state of roe deer organisms after the hot summer of 2015 compared to data from other studied periods.

## 5. Conclusions

In summary, our study shows that a warming climate—most likely due to heat stress and reduced food availability—may impact the reproductive potential of roe deer. We suggest that in the long-term life history, not only diapause but also the presence of the autumn rut are adaptations that allow roe deer to reproduce under optimal environmental conditions. In this situation, we propose that data describing the population and resource availability should be compared with climate data on a larger scale. Interdisciplinary research is essential today to optimize the management of animal populations and their habitat, which are subject to diverse changes.

## Figures and Tables

**Table 1:** Results of PERMANOVA test for constraints in redundancy analysis based on morphometric characteristics, using 999 permutations.

Variable	Df	Variance	F	Pr (>F)
Season	1	0.336	2.232	0.019
Year	1	0.838	5.568	0.001
Body mass	1	1.693	11.257	0.001
Age	1	0.537	3.567	0.002
Season × Year	1	0.403	2.676	0.009
Residual	101	15.194		

Abbreviations: Df—degrees of freedom; F—test statistic; Pr (>F)—*p*-value.

**Table 2:** Reproductive activity and fertility of female roe deer in western Poland, assessed based on the presence of corpora lutea (CL) on ovaries examined during different periods in the hunting years 2015/2016 and 2016/2017.

Season	2015/2016				2016/2017			
	Female		Number of All Observed CL		Female		Number of All Observed CL	
	N	After Ovulation	Total	Per One ♀	N	After Ovulation	Total	Per One ♀
autumn	20	8	9	1.1	31	31	60	1.9
winter	38	37	71	1.9	34	33	62	1.9
Total	58	45	80	1.8	65	64	122	1.9

Abbreviations: N—number of hunter-harvested individuals.

**Table 3:** Generalized Linear Mixed-Effects Models of follicle and corpora lutea numbers and the probability of their presence.

Response	Variable	Estimate	SE	z	Pr (>|z|)
Number of Corpora Lutea AICc = 258.0; AICc_0_ = 307.0; RE SD < 0.0001 R^2^_m_ = 0.449, R^2^_c_ = 0.449	(Intercept)	−1.171	0.290	−4.036	0.000
	Body mass	0.040	0.014	2.832	0.005
	Age	0.034	0.020	1.730	0.084
	Season = winter	1.021	0.175	5.824	0.000
	Year = 2016	1.029	0.172	5.997	0.000
	Season = winter × Year = 2016	−1.059	0.194	−5.451	0.000
Presence of Corpora Lutea AICc = 60.3; AICc_0_ = 89.6; RE SD < 0.0001 R^2^_m_ = 0.894, R^2^_c_ = 0.894	(Intercept)	−1.114	2.517	−0.443	0.658
	Body mass	−0.009	0.154	−0.059	0.953
	Age	0.293	0.207	1.419	0.156
	Season = winter	3.534	1.172	3.016	0.003
	Year = 2016	23.510	19,890.000	0.001	0.999
	Season = winter × Year = 2016	−23.450	19,890.000	−0.001	0.999
Number of Follicles AICc = 381.2; AICc_0_ = 385.4; RE SD < 0.0001 R^2^_m_ = 0.043, R^2^_c_ = 0.043	(Intercept)	−0.621	0.497	−1.250	0.211
	Body mass	0.062	0.030	2.026	0.043
	Year = 2016	−0.387	0.167	−2.314	0.021
Probability of Follicle Presence AICc = AICc_0_ = 168.4; RE SD = 0.2674 R^2^_m_ = 0.000, R^2^_c_ = 0.017	(Intercept)	0.439	0.232	1.891	0.059
				

Abbreviations: SE—standard error; z—test statistic; Pr (>|z|)—*p*-value; RE SD—standard deviation of forest inspectorate-specific random effect; AICc—Akaike’s Information Criterion; corrected for small sample size; AICc_0_—AICc of null (random effect and intercept-only) model; R^2^_m_—marginal coefficient of determination (amount of variance explained by fixed effects only); R^2^_c_—conditional coefficient of determination (amount of variance explained by both fixed and random effects).

## Data Availability

Data is contained within the article or Supplementary Material. The original contributions presented in the study are included in the article/supplementary material, further inquiries can be directed to the corresponding author/s.

## Supplementary Materials

The following supporting information can be downloaded at https://www.mdpi.com/article/10.3390/ani14213078/s1: Table S1: Features in the reproductive system of roe deer females in hunting years 2015/16 and 2016/17; Table S2: Features in ovaries of females lacking corpora lutea in autumn 2015.
